# Remote Sensing of Climatic Anomalies and West Nile Virus Incidence in the Northern Great Plains of the United States

**DOI:** 10.1371/journal.pone.0046882

**Published:** 2012-10-05

**Authors:** Ting-Wu Chuang, Michael C. Wimberly

**Affiliations:** 1 Geographic Information Science Center of Excellence, South Dakota State University, United States of America; 2 Department of Parasitology, College of Medicine, Taipei Medical University, Taiwan; 3 Center for International Tropical Medicine, College of Medicine, Taipei Medical University, Taiwan; Centers for Disease Control and Prevention, United States of America

## Abstract

The northern Great Plains (NGP) of the United States has been a hotspot of West Nile virus (WNV) incidence since 2002. Mosquito ecology and the transmission of vector-borne disease are influenced by multiple environmental factors, and climatic variability is an important driver of inter-annual variation in WNV transmission risk. This study applied multiple environmental predictors including land surface temperature (LST), the normalized difference vegetation index (NDVI) and actual evapotranspiration (ETa) derived from Moderate-Resolution Imaging Spectroradiometer (MODIS) products to establish prediction models for WNV risk in the NGP. These environmental metrics are sensitive to seasonal and inter-annual fluctuations in temperature and precipitation, and are hypothesized to influence mosquito population dynamics and WNV transmission. Non-linear generalized additive models (GAMs) were used to evaluate the influences of deviations of cumulative LST, NDVI, and ETa on inter-annual variations of WNV incidence from 2004–2010. The models were sensitive to the timing of spring green up (measured with NDVI), temperature variability in early spring and summer (measured with LST), and moisture availability from late spring through early summer (measured with ETa), highlighting seasonal changes in the influences of climatic fluctuations on WNV transmission. Predictions based on these variables indicated a low WNV risk across the NGP in 2011, which is concordant with the low case reports in this year. Environmental monitoring using remote-sensed data can contribute to surveillance of WNV risk and prediction of future WNV outbreaks in space and time.

## Introduction

West Nile virus (WNV) first appeared in the northeastern United States in 1999, and spread westward and southward after 2002 [1]. Subsequently, WNV has been reported in every state in the conterminous U.S. and become one of the most important vector-borne diseases due to its high human morbidity and impacts on avian populations [2], [3]. In the northern Great Plains (NGP), WNV caused a significant outbreak in 2003, and since that time the region has remained a major hotspot for human disease [4], [5]. Although relative spatial patterns of WNV risk in the NGP have remained relatively stable over time, there has been considerable interannual variability in human WNV incidence, with disease outbreaks often occurring in different locations in different years [6]. Climatic variability is known to have strong influences on the dynamics of virus circulation, vector abundance, and avian communities, and thus may explain historical fluctuations of WNV risk in the NGP [7]–[9]. Understanding the climatic determinants of WNV disease can also provide a basis for forecasting future disease risk based on lagged responses to antecedent environmental conditions.

Many studies have addressed the associations of WNV incidence with static environmental variables, including human demographics, physiography, land cover characteristics, land use practices, and the built environment [10]–[12]. In the NGP, high temperatures from May through July, intermediate precipitation from May through July, rural populations, and irrigated croplands were associated with high WNV incidence during the 2003 epidemic [6]. At a landscape scale, spatial patterns of human WNV cases in South Dakota were found to be associated with low elevations, rural areas, and poor soil drainage [13]. These associations are hypothesized to reflect the habitat preferences of the key mosquito vector, *Culex tarsalis* [9], [14], [15], although geographic variability in avian host communities is likely important as well.

In contrast, temporal variability of WNV incidence is likely to be affected by inter-annual climatic fluctuations. These climatic influences on WNV transmission risk have been studied in several regions of the United States. Higher temperatures were found to be associated with the epicenters of WNV activity between 2002 to 2004 in the U.S. [16]. In Mississippi, county-level WNV human risk was negatively associated with rainfall in the previous year [17]. Hydrological conditions were associated with the incidence of human WNV cases through their influences on vector abundance in Colorado [18]. Mosquito infection rates exhibited lagged responses to both temperature and precipitation in the Chicago region [19]. Although these studies have demonstrated the WNV incidence can be affected by climatic variability, most research to date has focused on small geographic areas or considered relatively short time periods.

The ecology of vector populations is a critical pathway through which environmental variability influences virus transmission and ultimately disease risk to humans. In temperate areas, the transmission of WNV requires virus amplification in avian hosts during the early spring and then spills over to dead-end hosts, like humans or horses, during the summer. In the NGP, mosquito abundance is highly synchronized with seasonal climatic fluctuations and can influence both amplification and transmission to humans [9]. The composition of bird communities also varies interannually in response to climate anomalies [20] and thus provides another pathway through which environmental variability can influence WNV risk. Temperature fluctuations also directly affect rates of virus amplification by influencing the extrinsic incubation period of WNV in the mosquito vector [16]. Thus, there are multiple mechanisms through which climate and other time-varying environmental drivers may influence interannual variability of WNV incidence in the NGP.

Environmental monitoring data obtained through satellite remote sensing has been widely used to assess the risk of vector-borne diseases over the past decade [21], [22]. Remotely-sensed data provide nearly seamless coverage across large spatial extents, affording new opportunities for investigating the environmental determinants of disease risk across regions like the NGP. Various environmental parameters relevant to mosquito-borne disease ecology can be derived from satellite imagery. For example, the Moderate-Resolution Imaging Spectroradiometer (MODIS) normalized difference vegetation index (NDVI) and land surface temperature (LST) products were used along with precipitation estimates from the Tropical Rainfall Monitoring Mission (TRMM) to predict spatial and temporal variability in malaria cases in Afghanistan [23]. Vegetation indices have also been used to identify habitats for WNV-competent vectors in urban areas and link them to potential transmission risk [24]. To date, however, remotely-sensed environmental indicators have not been used to model and predict inter-annual variability in WNV risk across large geographic areas.

To help remedy this knowledge gap, this study used multiple environmental variables derived from satellite remote sensing data to model county-level incidence of human WNV disease in the NGP over seven years (2004–2010). Our overarching hypothesis was that measurements of accumulated temperature and moisture throughout the growing season would serve as indicators of the environmental potential for WNV amplification in birds and transmission to humans, and would therefore be correlated with inter-annual fluctuations in the number of human WNV cases. We further hypothesized that the fit of models would improve as more data from later in the growing season were included, and that sensitivity to different environmental variables would change throughout the season. We also explored the potential for using these models to forecast WNV risk in late summer based on environmental conditions in spring and early summer.

## Materials and Methods

### Study Areas and WNV Human Cases

This study included seven States in the north-central U.S: Iowa (IA), Minnesota (MN), Montana (MT), Nebraska (NE), North Dakota (ND), South Dakota (SD), and Wyoming (WY). These states encompass the entire northern Great Plains (NGP) and its surrounding ecoregions. The NGP is characterized by low-relief landscapes dominated by prairie, grassland, and rain-fed agricultural fields. The weather varies throughout the year with cold winters, hot summers, and strong winds. The population density is much lower than the U.S. average (21.73 vs. 88.08/per square mile, Census 2010). Most counties in the NGP are classified as rural or mixed rural settings [25].

WNV positive human case data from the Centers for Disease Control and Prevention (CDC) were obtained from a website supported by United States Geological Survey (USGS) (diseasemaps.usgs.gov). The total number of county-level WNV neuroinvasive cases and WNV fever cases from 2004 to 2010 was used as the dependent variable in the study. Human WNV incidence during the initial outbreak years in 2002 and 2003 was likely affected by immunologically naïve avian and human populations and dispersal limitations. In order to focus on inter-annual climatic influences, we analyzed the subsequent years during which WNV was assumed endemic. Positive human cases met the CDC neuroinvasive and non-neuroinvasive domestic arboviral disease case definition and which included one or more clinical criteria and one or more laboratory criteria. Although reporting bias of WNV non-neuroinvasive cases is a potential concern for this type of passive surveillance system, a strong correlation between total reported WNV cases and neuroinvasive cases was reported in a previous study [26]. Thus, total numbers of reported WNV cases can capture temporal variability similar to that of neuroinvasive cases while allowing for a larger sample size.

### Environmental Predictors

We examined three environmental variables as predictors of WNV risk: land surface temperature (LST), the normalized difference vegetation index (NDVI) and actual evapotranspiration (ETa), all of which were derived from the Moderate Resolution Imaging Spectroradiometer (MODIS) remote sensing products. LST and NDVI have been used to analyze vector-borne diseases in many previous studies [23], [27], [28]. ETa is a hydrological variable measuring the flux of water from ground to atmosphere via evaporation and transpiration and is thus an indicator of moisture available at near the ground surface. ETa has been found to be associated with the abundance of *Ae. vexans* in New York [29] and temporal variability in malaria cases in Ethiopia [30]. In contrast to rainfall, ETa provides a more direct measurement of water availability on the ground which may serve as a more proximal indicator of mosquito breeding habitats.

MODIS data products at 1 km spatial resolution were acquired from Land Process Distributed Active Archive Center (LP DAAC). The mean LST was averaged from the day and night land surface temperatures derived from the MODIS Terra land surface temperature and emissivity product (MOD11A2). The NDVI was calculated from the band 1 (visible) and band 2 (near infrared) of the MODIS combined TERRA/AQUA nadir BRDF-adjusted reflectance product (MCD43B4) using the equation below.
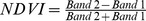



Actual evapotranspiration (ETa) was calculated based on Famine Early Warning System Network (FEWS-NET) potential evapotranspiration (PET) and elevation-corrected LST using the simplified surface energy balance method [31], [32]. All these indices were aggregated and summarized at county-level using the Zonal Summary tool in ArcGIS 9.3 (ESRI, Redlands, CA). LST and ETa were summarized for 8-day composite periods, whereas NDVI was summarized over a 16-day composite period that was updated at 8-day intervals. For consistency, the last date of each composite period was used as the composite date. For example, the April 22 LST composite included data from April 15-April 22, whereas the April 22 NDVI composite included data from April 7-April 22.

In the NGP, the transmission pattern of WNV is highly seasonal, and human cases are usually reported during summer and early fall. In South Dakota, for example, more than 90% of human cases occur after July 1. Laboratory evidence has shown that 14.3°C is the threshold temperature for the activity of WNV [16], and we assumed that there was minimal mosquito vector and virus activity during winter in the NGP. Therefore, we only included environmental predictors from early spring to summer in our statistical model to predict the incidence during the summer of the same year. The start date of the first MODIS composite was April 7 and the end date of the last MODIS composite was August 12.

### Statistical Analysis

The purpose of our analysis was to evaluate the contributions of different seasonal environmental variables for predicting the occurrence of WNV outbreaks in NGP. In order to minimize the influences of unstable incidence rates from rural counties with extremely low populations, we applied several approaches to transform the raw WNV human incidence rate to more reliable indicators. The spatial empirical Bayes (SEB) smoothed rate was used to map spatial patterns of the annual WNV incidence in the NGP throughout 2004–2010 for exploratory visualization. This spatial smoothing technique uses empirical Bayes methods to borrow strength from neighboring counties and minimize problems associated with small populations at risk [33], [34]. The SEB smoothed rate was calculated using GeoDa 0.95 [34].

In the statistical models, we included only the 66 counties with ten or more cumulative WNV cases during the study period to focus on areas with the highest potential for measuring temporal variability in risk to humans. We calculated the logarithm of relative risk (LRR) to replace the raw incidence rate for each county in each year. The LRR quantified the interannual variability of WNV incidence for each county and was calculated using the following equation.
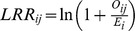
where *i* indexes counties, *j* indexes years, *O* = observed cases, and *E* = expected cases.

Three steps were used to calculate the deviation percentage of each environmental variable to quantify annual anomalies relative to the long term average. First, cumulative environmental indices (CENV) were calculated for LST, NDVI and ETa for every composite date in every year. A threshold temperature of 14.3°C was set to calculate the growing degree day of LST (cumulative LST) based on the threshold temperature for virus activity [16]. A threshold value 0.25 was set to calculate cumulative NDVI to avoid the background noise resulting from snow cover in early spring. No threshold value was set for cumulative ETa. Second, the 10-year average (2002–2011) of each of the three cumulative indices (MCENV) was estimated for each composite date in each county.
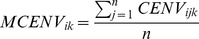
where *k* indexes the starting date of each composite period and *n* = number of years.

Finally, the deviance percentage of each cumulative environmental predicator (DCENV) was calculated as a function of the annual indices and their 10-year averages.
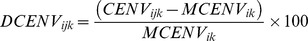



Generalized additive models (GAMs) were applied to analyze the associations between the percent deviations of environmental predictors throughout spring to summer and annual relative risk of WNV. The GAMs used a spline smoothing operator which allows fitting data with nonlinear relationships [35]. We used the data from 2004 to 2010 to fit the models and tested the forecast of relative risk in 2011. The generated form of the GAM models was
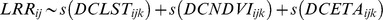
where *s* represents a spline smoothed function of the independent variable.

A total of 15 sets of models were fitted, each corresponding to a specific date ranging from April 22 to August 12 at 8-day intervals. These model dates represented the latest composite date of the cumulative environmental indices that could be included in each model. For each model date, there were multiple candidate models that included all combinations of the three climate variables summarized for all composite dates at or earlier than the model date. The models were evaluated using the Akaike’s Information Criterion (AIC) and the model with the lowest AIC was selected as the best fit for each date [36]. This approach allowed us to select a best predictive model for each model date and determine whether model fit improved as additional environmental information from later in the season was incorporated. We applied a cross validation to assess the predictive capabilities of the models by dropping one year at a time, fitting the model with the remaining years, predicting LRR based on the environmental variables for the year that was withheld, and computing the root mean square error (RMSE) to evaluate the prediction accuracy.

Statistical modeling and model validation were carried out using R 2.13 (R Development Core Team 2011) and the mgcv package was used for the statistical modeling. Two models (June 25 and July 27) were selected to generate the forecasting maps of WNV risk for 2011. These two models dates were selected to assess an early-season prediction versus a late-season prediction. Forecasting maps were generated in ArcGIS 9.3 (ESRI, Redlands, CA) by applying the models to all counties within the NGP.

## Results

**Figure 1:**
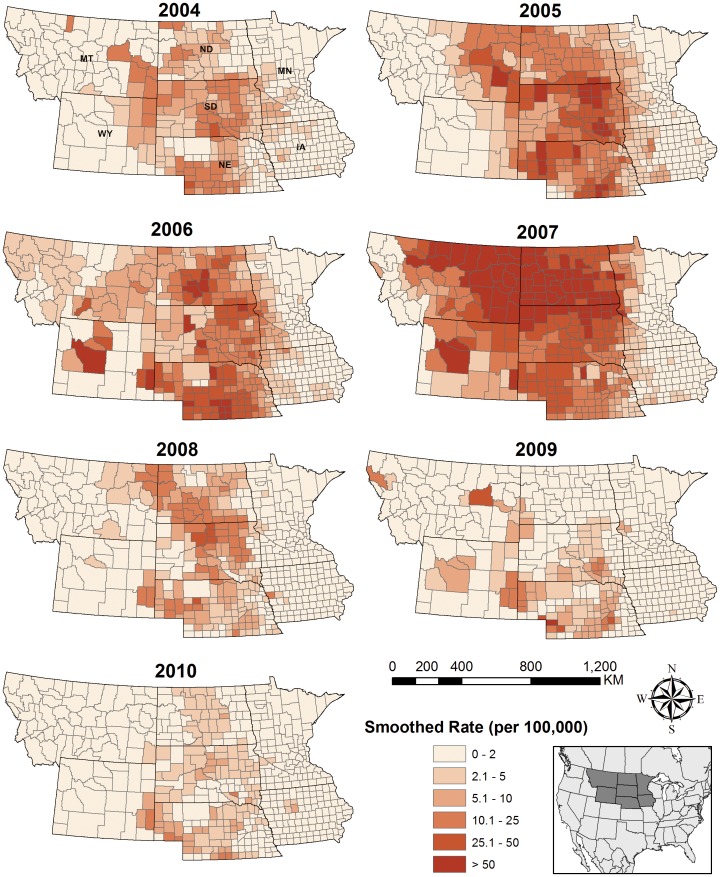
Spatial empirical Bayes (SEB) smoothed WNV Incidence Rate in the Northern Great Plains throughout 2004 to 2010.

Annual WNV incidence rates were compared between the 7-state NGP region and the entire United States in Table 1. The incidence rates were significantly higher in the NGP than the nation as a whole, and nearly 20% of cases in the U.S. from 2004 to 2010 occurred in the NGP. The particularly high 2007 WNV incidence rate in the NGP was not observed at the national level, suggesting that environmental changes at the regional level are one of the important drivers. The SEB smoothed incidence rates revealed spatial variability of the annual WNV incidence rate within the NGP (Figure 1). The counties with highest incidence were usually located within ND, SD, and NE. The areas with highest incidence were generally in the Glaciated Plains ecoregions on the east side of Missouri river. In contract, the forest and cropland-dominated areas in Minnesota and Iowa and mountainous regions in Montana and Wyoming tended to have lower incidence rates. However, the 2007 outbreak demonstrated an unusual expansion of WNV west of the Missouri river into the northwestern Great Plains, whereas in 2009 WNV was more concentrated in the southern part of the region. From 2007 to 2010 WNV incidence decreased in the NGP, although moderate to low level incidence was observed in portions of ND, SD, and NE.

**Table 1:** Comparisons of WNV incidence rate between the NGP and the U.S. throughout 2004 to 2010.

Year	NGP		U.S.		Percentage of Cases (NGP/U.S.)
	Human Cases	Incidence*	Human Cases	Incidence	
2004	197	1.60	2539	0.91	7.76%
2005	622	5.04	3000	1.07	20.73%
2006	715	5.79	4269	1.53	16.75%
2007	1254	10.15	3630	1.30	34.55%
2008	152	1.23	1356	0.48	11.21%
2009	100	0.81	720	0.26	13.89%
2010	91	0.74	1021	0.37	8.91%
Total	3131	25.35	16535	5.91	18.94%

* per 100,000.

**Figure 2:**
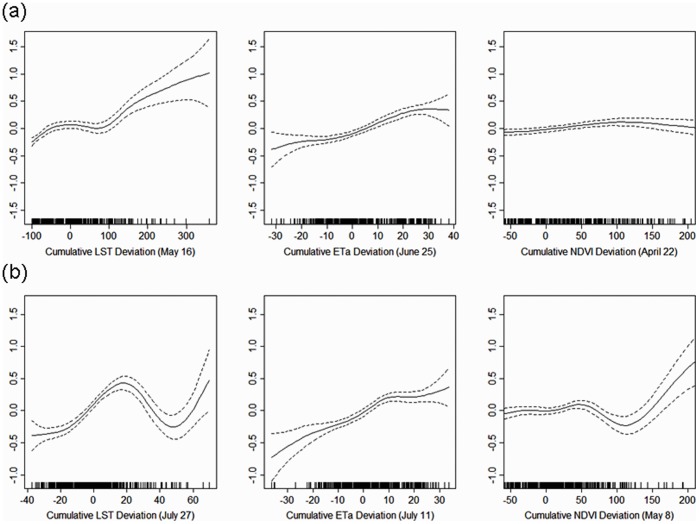
The plotting of GAM best-fitting results (a) Model Date: Jun 25 (b) Model Date: Aug 12 between WNV risk and environmental variables.

Generalized additive models for each period from April 7 to August 12 incorporated environmental predictors prior to each model date. The best-fitting models with the lowest AIC values for each model date are listed in Table 2. The decreasing AIC and increasing adjusted R-square values indicated that the prediction power of models gradually increased as environmental data were accumulated through spring and into summer. Cumulative LST, ETa, and NDVI generally showed positive associations with WNV relative risk (Figure 2). However, WNV relative risk was highest at intermediate LST deviations in the late season models, suggesting that WNV is associated with a climatic niche rather than increasing monotonically with temperature.

**Table 2:** GAM Model selection results for each model date.

No.	Model Date*	The composite date of environmental variables in the best-fitting models			AIC	Adjusted R^2^
		LST	ETa	NDVI		
1	April 22	April 22	April 22	April 22	549.7	17.7
2	April 30	April 30	April 30	April 22	522.2	29.6
3	May 8	May 8	April 30	May 8	488.2	37.8
4	May 16	May 16	April 30	April 22	453.1	44.6
5	May 24	May 16	May 24	April 22	438.3	39.7
6	June 1	May 16	June 1	April 22	432.3	40.8
7	June 9	May 16	June 9	April 22	426.2	41.9
8	June 17	May 16	June 17	April 22	410.1	44.2
9	June 25	May 16	June 25	April 22	391.7	46
10	July 3	May 16	July 3	April 22	375.1	48
11	July 11	May 16	July 11	April 22	348.1	51.6
12	July 19	July 19	July 11	May 8	323.6	58.7
13	July 27	July 27	July 11	May 8	303.3	60.5
14	August 4	August 4	July 11	May 8	280.4	62.5
15	August 12	August 4	July 11	May 8	280.4	62.5

* Each model was fitted using environmental variables between the earliest composite date (April 22) and the model date.

Examining the composite dates of the cumulative environmental indices included at each model date showed how the sensitivity of WNV to these variables changed throughout the season. Cumulative LST through composite date May 16 and cumulative NDVI through composite date April 22 remained in the models through model date July 11 (Table 2). After this model date, the cumulative LST anomalies through composite date July 19 and later were included in the models. Between model dates May 17 and July 3, model fit increased as cumulative ETa was totaled over longer portions of the growing season.

The latest composite date for cumulative NDVI in any of the models was May 8, indicating that WNV risk was only sensitive to cumulative NDVI early in the growing season. In contrast, WNV risk was sensitive to change in cumulative LST early in the growing season (composite dates April 22-May 16) and again later in early to mid-summer (composite date July 19 and later). Sensitivity to increasing cumulative ETa was greatest during the late spring and early summer (composite dates May 24-July 11) and weaker in mid to late summer (composite date July 11 and later).

**Figure 3:**
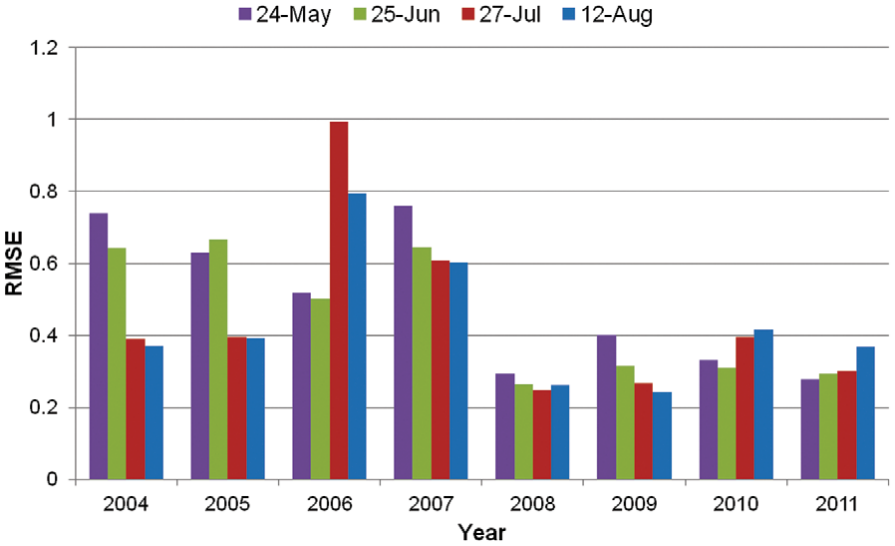
Cross-validation of generalized additive models for four model dates from 2004–2010. The RMSE results for 2011 are a validation based on the model fitted with data from 2004–2010.

The results of cross-validation from 4 model dates (May 24, June 25, July 27, and August 12) are shown in Figure 3. Overall, the models showed better prediction for low risk years (2008–2010) than moderate-to-high risk years (2004–2007). In 2004 and 2005, predictions were more accurate for the later season models (July and August), whereas in 2006 predictions were more accurate for the early season models (May and June). Seasonal differences in prediction accuracy were comparatively small from 2007–2010.

**Figure 4:**
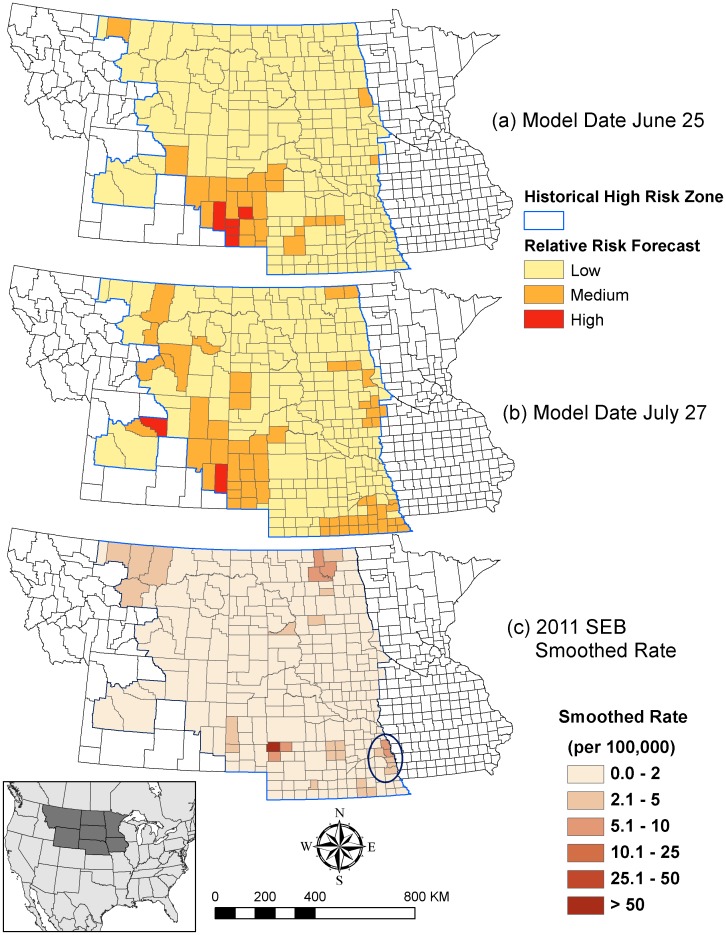
WNV relative risk forecasting maps for 2011 at the (a) model date June 25 and (b) model date July 27 and (c) 2011 SEB smoothed WNV incidence rates. The circle indicates the Omaha-Council Bluffs metropolitan area.

Two WNV risk forecasting maps in 2011 were generated by the best fitting models for model dates June 25 and July 27 (Figure 4). Predictions were limited to the historical zone of high risk WNV within the NGP, which included all the counties in ND, SD, and NE and other counties with smoothed SEB incidence rates equal or larger than 8.5 WNV cases per 100,000 per year for 2002 to 2010. Because our analysis criteria excluded the counties with cumulative WNV human cases less than 10 in the study period, most of the counties included in the statistical models are within this zone. For this reason, our forecasting results in 2011 were only shown within this zone to avoid over-extrapolating the predictions. Both risk maps indicated that the WNV risk in 2011 was at relatively low across most of the NGP. This result is concordant with the CDC WNV surveillance data [37] and the 2011 SEB smoothed incidence rate (Figure 4). In 2011, only 50 WNV human cases were reported from the NGP and the overall incidence rate was 0.40 per 100,000.

Some, but not all of the areas with higher incidence in 2011 fell within the moderate-to-high risk counties predicted by the model, A relatively high proportion of the 2011 cases (22) occurred in eastern NE within Omaha-Council Bluffs (population of 877,000 in 2011), the largest metropolitan area within the historical high-risk zone (Figure 4). Of the counties in this metropolitan area, only Sarpy County NE exhibited a distinctive increase in WNV cases (8 cases in 2011 compared to a maximum of 3 cases per year from 2004–2010). Sarpy County was identified as a low risk county in the forecast for model date June 25, but was classified as moderate-risk in the later-season forecast for model date July 27.

## Discussion

This study applied environmental variables derived from satellite remote sensing data to model WNV transmission risk in the NGP. The forecast for 2011 was generally accurate at a regional level, indicating that at least some of the interannual variability in WNV transmission can be captured by remotely-sensed variables such as land surface temperature, actual evapotranspiration, and the normalized difference vegetation index. This finding also supported our overarching hypothesis that these environmental predictors influence a variety of ecological processes which affect the potential for virus amplification and ultimately transmission to humans. However, the cumulative environmental indices may also mask short-term environmental variability, such as heavy rains that wash out breeding sites or cold periods that interrupt WNV transmission. Continued model updating, prediction and evaluation will be necessary to assess forecasting skill under changing future conditions.

The different temporal responses of each environmental variable also revealed how associations between interannual variability of WNV incidence and deviations of cumulative LST, NDVI, and ETa varied throughout the season. WNV relative risk was sensitive to cumulative LST in early spring, as evidenced by the fact that cumulative LST through composite date May16 was included in most of the prediction models with model dates in spring and early summer. Thus, early spring temperature conditions may be particularly important for reducing the extrinsic incubation period (EIP) and triggering virus amplification in the avian communities. On the other hand, *Culex* species tend to draw blood meals from avian species in spring and early summer and shift to take blood meals from humans in summer and fall [38]. This shifting behavior facilitates virus transmission from the avian community to other hosts, including humans. Bell et al. (2006) proposed a similar idea in a study in North Dakota and Minnesota which showed the higher temperature was associated with a longer WNV transmission season, higher mosquito infection rates, and higher numbers of human cases [39].

NDVI is metric of vegetation greenness with a seasonal pattern that is mainly sensitive to temperature, but also to moisture conditions in the NGP. In our prediction models, cumulative NDVI through April to early May served primarily as an indicator of the timing of the onset of spring and emerged as a predictor of WNV relative risk. Early onset of spring indicates that the environmental conditions are suitable for vegetation growth and also for earlier emergence of mosquito populations. In the NGP, the time available for virus amplification in the enzootic cycle is critical because the extremely cold winter in this region makes the mosquito season shorter than warmer areas. Small scale virus circulation in mosquito and avian populations during the winter has been shown in California but seems unlikely in the NGP [40]. Thus, a longer mosquito season increases the time available for virus transmission and amplification in avian communities prior to the onset of human transmission during the summer.

Actual evapotranspiration is an important hydrological process that encompasses water movement from the land surface to atmosphere through the combined processes of evaporation from the soil surface and transpiration of subsurface moisture by plants. A positive ETa can indicate higher-than-average temperature or soil moisture availability, both of which are expected to have positive relationships with WNV risk. The improvement in model fit as additional information on ETa was accumulated from model dates May 24 through July 11 suggests that the sensitivity of WNV risk to surface moisture and mosquito breeding habitats is strongest during this part of the season. In contrast, improvements in fit for model dates of July 19 and later were driven by cumulative LST, suggesting that temperature is an important climatic risk factor during the summer as WNV transmission shifts from birds to humans.

Multi-temporal models incorporating these three remotely-sensed environmental variables fit the data well and produced reasonable forecasting results. Models with dates of June 1 and later explained between 40–62% of the spatial and temporal variability in relative risk. Model fit increased later in season as the time between the model prediction and the main WNV transmission season grew shorter, reflecting the expected trade-off between lead time and accuracy of ecological forecasts. The remaining unexplained variability likely reflects numerous other drivers of WNV risk that were not included in our model, such as WNV seroprevalence in avian community, changes in avian community composition over time, vector control, and human behavior. The passive disease surveillance data that we used also has numerous sources of uncertainty, which further reduced our ability to model WNV risk as a function of environmental variables Even so, the model still produced a reasonable prediction of the regional patterns of WNV risk in 2011.

The lagged effects of warmer temperature and higher precipitation on increasing human WNV incidence have been demonstrated in a previous study [41]. This study summarized meteorological conditions using weather station data. However, the availability and distribution of stations may not represent spatial and temporal climatic variations well, especially in the rural areas. Our study took advantage of satellite remote sensing data sources to develop multiple prediction models based on the environmental conditions. Earth-observing satellites provide timely and consistent repeated measurements across most of the earth’s land surface, which are valuable for monitoring and forecasting disease risk at broad regional scales [42], [43]. We also demonstrated how the sensitivity of WNV incidence to different environmental variables changes throughout spring to summer.

The national WNV outbreak appears to have declined from 2007 through 2011 according to surveillance reports [4]. However, localized outbreaks still can be observed in specific locations. For instance, in 2010 there was a major WNV outbreak in Arizona [44], [45] and a resurgence of cases in parts of the New York City metropolitan area [3]. Recently, many WNV outbreaks have also been reported in many countries in Europe, including Romania, Italy, Greece, and Spain. [46]–[49]. The possibility of continued evolution of WNV is also a concern. In the U.S., the original WNV strain (NY99) was replaced by a more virulent strain (WN02) with a shorter extrinsic incubation time in the mosquito vector [50], [51]. Furthermore, a national serological survey of blood donors between 2003 and 2008 also showed the low cumulative prevalence (1%) of past WNV infection in the U.S. [52]. Thus, a large proportion of the population is still susceptible to the risk of WNV infection. This evidence highlights the potential for future outbreaks, which will likely occur at times and in places where climatic conditions are most suitable for disease reemergence.

The continuing risk of WNV outbreaks poses a number of challenges for WNV surveillance. For example, passive surveillance based on voluntary reporting of dead birds will likely become less effective if concerns about WNV risk fade from public attention [53]. Surveillance of mosquito populations or sentinel animals can be effective, but is likely to be highly concentrated in specific areas where sufficient resources are available. Surveillance of environmental risk factors based on environmental monitoring data offers a complementary approach that can highlight the places and times that are most at risk for future outbreaks. A goal for future research should be to blend information from these various types of surveillance data to improve forecasting via data assimilation and other advanced data-model fusion techniques [54].
